# Aesthetic emotions, what are their cognitive functions?

**DOI:** 10.3389/fpsyg.2014.00098

**Published:** 2014-02-11

**Authors:** Leonid Perlovsky

**Affiliations:** Athinoula A. Martinos Center for Biomedical Imaging, Harvard UniversityCharlestown, MA, USA

**Keywords:** emotions, aesthetics, beautiful, cognition, music, Kant, mind, mathematical models

## Difficulties of defining “aesthetic”

A huge number of publications are devoted to aesthetic emotions; Google Scholar gives 319,000 references. Nevertheless, finding a definition of what is aesthetic is not easy. Most authors use no definitions. Wikipedia (2013) gives a circular definition: “aesthetic emotions… are felt during aesthetic activity.” This is similar to the “institutional theory of art” that defines art as what is considered so by an accepted art institution (Dickie, 1974). In 70 years of discussions in the Journal of Aesthetic and Art Criticism this theory, despite its obvious flaws, remains accepted among philosophers of art. Scientists studying emotions should aspire for a more meaningful definition, yet it is not easy to find. For example, Juslin (2013) devotes a special section in his work introducing aesthetic emotions as a fundamental innovation of his theory of musical emotions, yet no definition of aesthetic is given.

Difficulties of contemporary theorists attempting to define “aesthetic” might be related to Kant (1790). He rejected an older idea that aesthetic is related to a special perception ability (Baumgarten, 1992/1750) and attempted to define aesthetic as related to knowledge. This article suggests that Kant came amazingly close to the contemporary scientific understanding, and it clarifies why he could not formulate this idea to his satisfaction. The best he could do is to say that aesthetic emotion is *disinterested*. On many pages he has repeated that this only concerns everyday mundane interests, that the beautiful is related to some of the most important human interests, that a better definition is needed, but “today” he could not give a satisfactory positive definition of what it is. From Schiller to this very day many discussions continue the false tradition of characterizing aesthetics and beautiful as disinterested (Wikipedia, 2013; Stanford Encyclopedia Stolnitz, 1960; Scruton, 1974, 2007; Guyer, 1997; de Sousa, 2013; Juslin, 2013; Zangwill, 2013; to name just a few among thousands).

This article defines aesthetic and the beautiful in correspondence with Kantian ideas, our deepest intuitions about the beautiful, the Aristotelian “unity in manifold,” (Aristotle, 1995) and in agreement with contemporary understanding of the neural mechanisms of emotions and cognition.

## Mathematical models of emotions and cognition

Here is a short summary of this complicated topic, which is an active area of research with thousands of publications; the mathematical model captures essential aspects of the mind mechanisms, it gives many predictions confirmed experimentally and does not contradict known data (more details and references can be found in Perlovsky et al., 2011). This summary is aimed at understanding the mental mechanisms of aesthetic emotions in the next section. Among the most ancient mind mechanisms are instinctual drives. According to the Grossberg and Levine (1987) theory of drives and emotions, instinctual drives can be modeled as internal sensors that measure vital bodily parameters and indicate their safe ranges. If a parameter is outside its safe range, this information is transmitted by neural signals to decision-making parts of the brain-mind initiating appropriate decisions and behavior. These neural signals are perceived internally as emotions motivating behavior. For example, we have sensors measuring the sugar level in blood, when it is below a certain level we feel it as hunger and devote more attention to finding food.

We perceive food and other objects by matching mental representations (memories) of objects to patterns in sensor signals (Kosslyn, 1994). Mental representations are organized into an approximate hierarchy (Grossberg, 1988) from perceptual elements, to objects, to contexts and situations, and higher up to abstract concepts. The evolutionary purpose of evolving the hierarchy is to enable abstract concepts. For example, the representation “professor office” unifies lower-level representations of objects (chair, desk, computer, books) into a unified concept of the office. Similarly, concepts of offices, lecture halls, etc. are unified into a concept of “university,” “educational system,” etc. A neural theory of how information-based pleasure arises from a network involving association cortex, dorsolateral prefrontal cortex, orbitofrontal cortex, striatum, opioids, and dopamine is outlined in Levine (2012). These detailed neural mechanisms of generalized mental representations are not yet accounted for in a simplified mathematical model of interacting emotions and cognition, which misses many details (Pessoa, 2008, 2009), but it is sufficient for the purpose of this article.

## The knowledge instinct, aesthetic emotions, and the beautiful

The Grossberg and Levine (1987) theory has been extended from bodily needs to learning (Perlovsky, 2001, 2006, 2007, 2008a, 2013a,b; Perlovsky et al., 2011). Satisfaction of bodily needs and our very survival requires understanding of the surrounding world. Therefore, possibly the most important instinct (for humans and higher animals) is an instinct for knowledge, driving learning, knowledge acquisition, and improvement of mental representations for better correspondence to the world. A mathematical model of the knowledge instinct has been discussed in the above references, and candidate neural mechanisms are discussed in Levine and Perlovsky (2008), Levine (2012), Perlovsky and Levine (2012). Similar to other instincts, satisfaction and dissatisfaction of this instinct are perceived emotionally. These specific emotions related to knowledge are called aesthetic emotions. An experimental proof of their existence has been given in Perlovsky et al. (2010).

At lower levels of the mental hierarchy these emotional neural signals are below the conscious threshold. We are not elated with aesthetic pleasure when recognizing an everyday object. However, when we do *not recognize* familiar objects or situations we immediately perceive these aesthetic emotions, we could become scared. This is one of the standard tricks of thriller movies. At higher levels of the hierarchy we may consciously perceive positive and negative aesthetic emotions. When one solves a problem he or she has been thinking about for a long time, one often feels positive emotions. This is not just a utilitarian emotion due to expecting a salary raise, or being closer to finishing a dissertation. One also feels aesthetic emotions due to satisfaction of the knowledge instinct. The “highest” aesthetic emotion of the beautiful is felt when the knowledge instinct is satisfied at the highest levels of the hierarchy.

## Aesthetic emotions and contents of the “highest” representations

Mental representations at every hierarchical level, as discussed, have an evolutionary purpose to unify lower level representations. The purpose of representations at the top of the hierarchy is to unify one's entire life experience. This unity is felt as the meaning of life; it is important for concentrating one's effort on the most meaningful aims, it is essential for survival, and for achieving the highest goals. For better understanding what this really means we have to go back and consider some details of learning mechanisms.

Mental representations are not as clear and crisp as perceptions of objects. Consider an object in front of your eyes, then close your eyes and imagine this object. The imagination is not as clear as the perception with opened eyes. Imaginations are produced by neural projections of representations to the visual cortex. Vagueness of imaginations testifies to the vagueness of representations. Vagueness of representations has been experimentally demonstrated in brain imaging experiments (Bar et al., 2006; Kveraga et al., 2007; Perlovsky, 2009c). In addition, it has been shown that vaguer representations are also less accessible to consciousness. It follows that abstract representations higher up in the hierarchy, which are based on multiple vague lower level representations, are vague and barely conscious. Their cognitive contents are mixed up with their emotional contents.

However, we can consciously and in detail discuss the meaning of life, and argue for or against its existence. Does this not contradict the above thesis about vagueness and unconsciousness of higher representations? No. And the reason is that language and cognition are separate systems; closely connected, but still separate. Even as we cannot clearly differentiate them in our subjective consciousness, mathematical models of interacting language, cognition, and emotions let us understand how they interact (Perlovsky, 2009a,b, 2013c). Predictions of these models, in particular that abstract concepts are vague, barely conscious, and are understood mostly due to language, are confirmed experimentally (Binder et al., 2005; Price, 2012).

The separateness of language and cognition explains why it is difficult to agree about the meaning of life and the aesthetic emotion of the beautiful. Because these ideas are at the top of the mental hierarchy they are so important (Kant, 1790), for this reason great thinkers for millennia have discussed them, cultures have developed them, and language makes this accumulated knowledge accessible to everyone. But because our subjective perceptions of these ideas are vague, doubts remain. There are no direct subjective conscious confirmations of these cultural constructs, and so far scientific evidence is limited. An important scientific challenge for the near future is to demonstrate that the beautiful is an aesthetic emotion related to satisfaction of the knowledge instinct at the top of the mental hierarchy. Cognitive representations near the top of the hierarchy are vague and unconscious, their contents are “veiled” from our consciousness by language, and therefore “measuring” emotions of the beautiful related to improving these contents is difficult. The beautiful is a rare emotion because the meaning of life is not learned like simple concepts. Most of us can hope for a rare experience confirming that the meaning really exists; at such a moment one experiences emotions of the beautiful.

## Multiplicity of aesthetic emotions

Human emotional life is rich; we can experience a huge number of emotions, possibly a continuum, not just a few for which we have words, like fear, sadness, joy, etc. The English language has about 150 emotional words, and among these only between 5 and 20 are appreciably different (Petrov et al., 2012). The most advanced scales for rating musical emotions still use emotional words for a few emotions (Zentner et al., 2008). But emotional experiences are much richer than just the few emotions found in English language. The diversity of emotions is most apparent when listening to music; virtually every musical phrase produces a new emotion. What is the origin and cognitive function of the multiplicity of emotions?

The knowledge instinct does not just maximize a single similarity between all representations (knowledge) and all sensor patterns; it acts at every level of the hierarchy, maximizing similarity between bottom-up and top-down signals. In addition it drives the mind to resolve contradictions between knowledge and instinctual drives, and between various elements of knowledge. These contradictions, known as cognitive dissonances (Festinger, 1957; Harmon-Jones et al., 2009), are perceived emotionally. There could be a degree of contradiction between any pair of representation-concepts, and every contradiction is potentially experienced as a separate emotion. People differ in perception of these contradictions and in categorization of corresponding emotions. Still here could be the foundation of a need for diverse emotions. Resolving cognitive contradictions requires abilities for the conscious experience of a large number of emotions. They are needed to maintain diverse knowledge in our minds and for the entire human evolution (see more detailed discussions in Perlovsky, 2008b, 2010, 2012a,b, 2013a). These emotions evolved along with language. As language vocalizations have been losing their emotionality, a separate ability for highly emotional vocalization evolved into music; the still remaining emotionality of language prosody is essential for the continued evolution of languages and cultures (Perlovsky, 2013a,b). Some of these theoretical predictions have been confirmed experimentally (Perlovsky et al., 2010, 2013; Bonniot-Cabanac et al., 2012; Masataka and Perlovsky, 2012, 2013; Cabanac et al., 2013).

## Future research challenges

Human emotional life is rich; we can experience a huge variety of emotions, most of which are aesthetic. The immediate challenge is to develop experimental techniques for measuring multiplicity of aesthetic emotions. One difficulty is that aesthetic emotions might be subjective and change over time for each individual depending on internal states and external circumstances (e.g., Chapin et al., 2010). Therefore, averaging over individuals often leads to losing fine emotional differentiation and to detecting the most ancient and robust aspects of emotions, valence and arousal. Another difficulty is the use of emotional words in most experimental studies (e.g., Eerola and Vuoskoski, 2011); words are not suitable for measuring emotions inexpressible in words, such as emotions in prosody and music, which evolved for a specific purpose to complement the emotional limitations of language. Detecting a large number of aesthetic emotions, in particular musical emotions, could be approached by subjective estimation of the differences among musical excerpts, and then applying multidimensional scaling to these measures.
